# Supplementary figures and images for: Transcriptomic and proteomic profiles of II YOU 838 (Oryza sativa) provide insights into heat stress tolerance in hybrid rice
Source: PeerJ. 2020 Feb 21;8:e8306. doi: 10.7717/peerj.8306 (PMC7039125; doi:10.7717/peerj.8306)

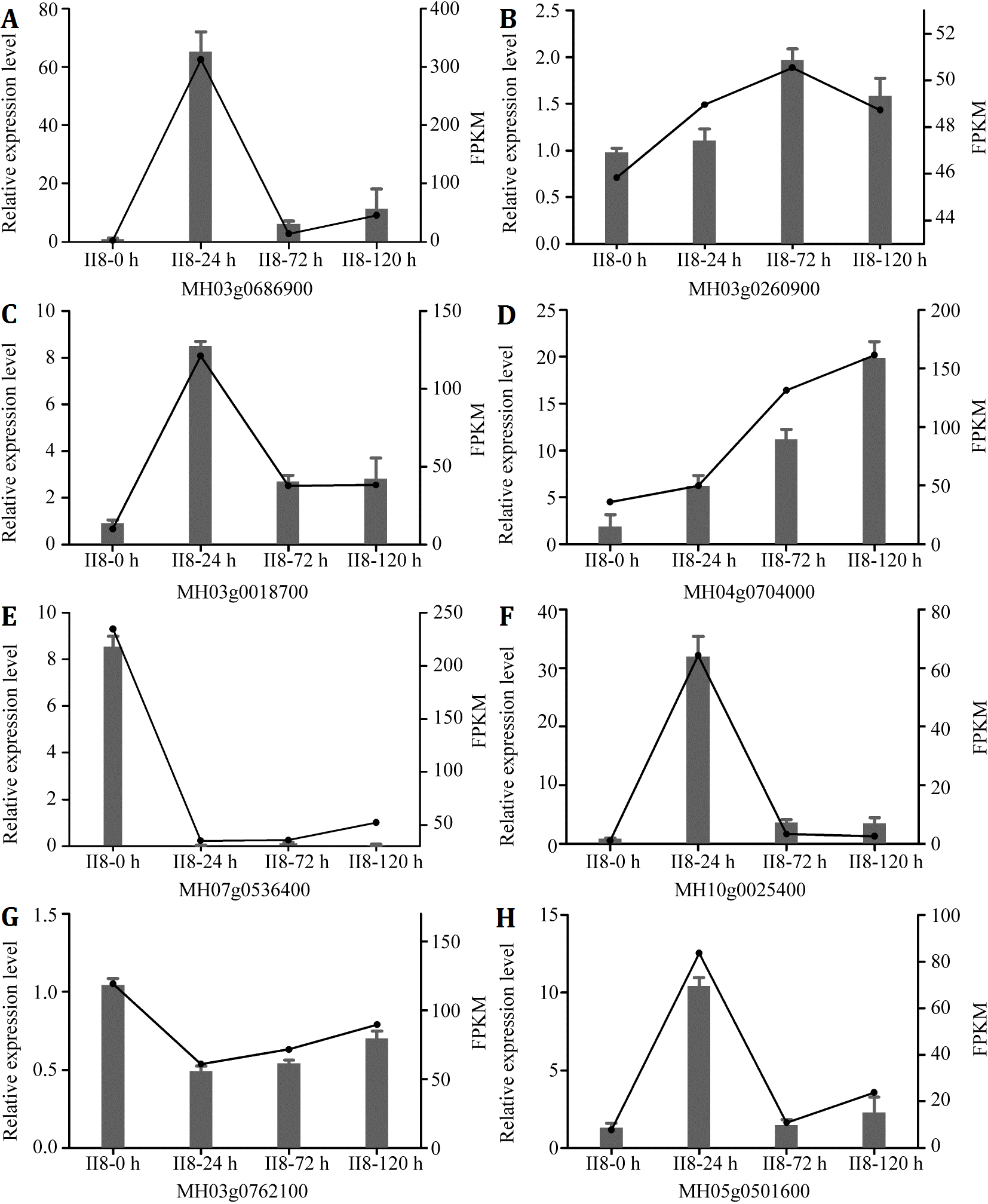

Supplement: Supplemental Information 1 — The columns indicate the relative expression levels obtained by qPCR and the lines indicate the FPKM values obtained by RNA-Seq. Error bars are standard deviations of the mean (n = 3) among the three repeats. The three internal control genes were MH03g0618400 (ACTIN1), MH01g0139700 (Mpv17), and MH02g0177100 (chloroplast processing peptidase). The candidate genes selected for validation are: (A) MH03g0686900, mitochondrial import inner membrane translocase subunit TIM14-3. (B) MH03g0260900, HUA2-like protein 2. (C) MH03g0018700, heat shock 70-kDa protein. (D) MH04g0704000, peroxidase 12. (E) MH07g0536400, peroxidase 2. (F) MH10g0025400, probable ascorbate-specific transmembrane electron transporter 1. (G) MH03g0762100, heat shock transcription factor gene OsHsfA1. (H) MH05g0501600, heat shock transcription factor gene OsHsfA4d. [file peerj-08-8306-s001.png]

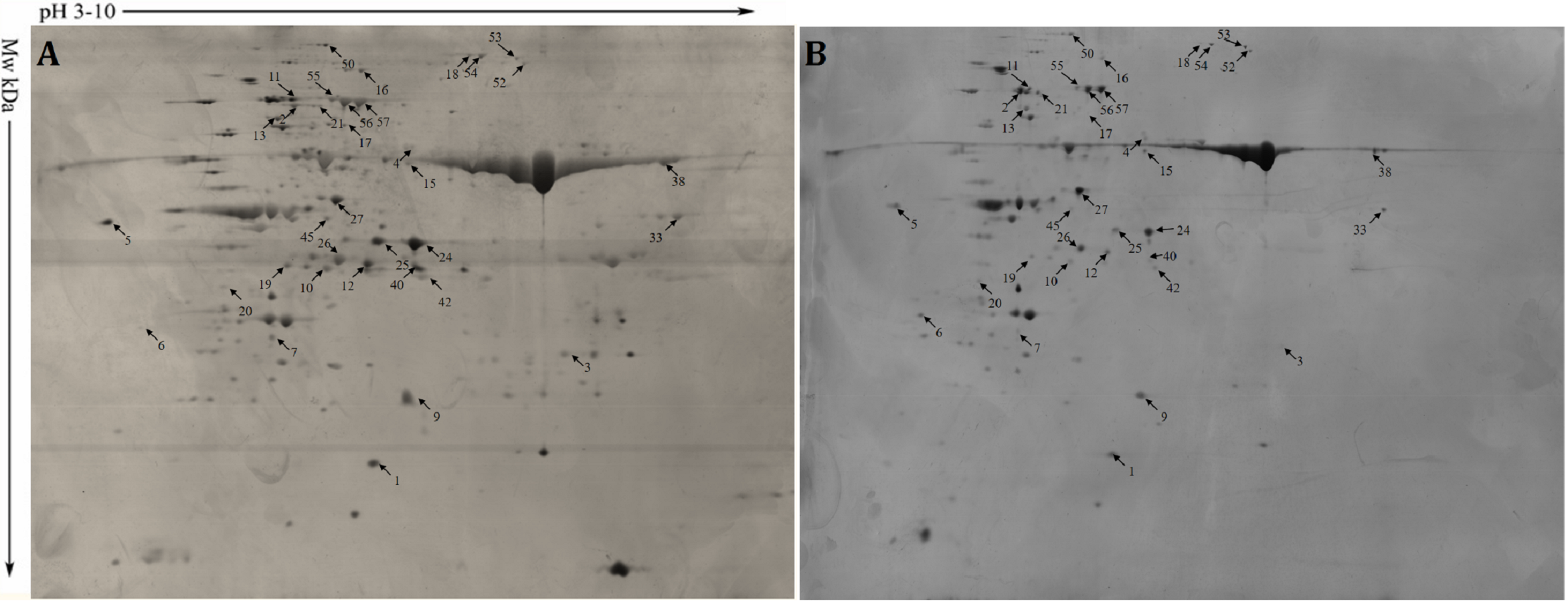

Supplement: Supplemental Information 2 — (A) Proteins extracted from II YOU 838 flag leaves prior to heat stress (0 h). (B) Proteins extracted from II YOU 838 flag leaves after 24 h of heat stress. The extracted proteins were separated by 2D-PAGE and fast silver stained. Arrows indicate the differentially abundant proteins. Relative abundance was analyzed using ImageMaster 5.0 software. The numbered spots are the differentially abundant proteins after 24 h of heat stress. [file peerj-08-8306-s002.png]

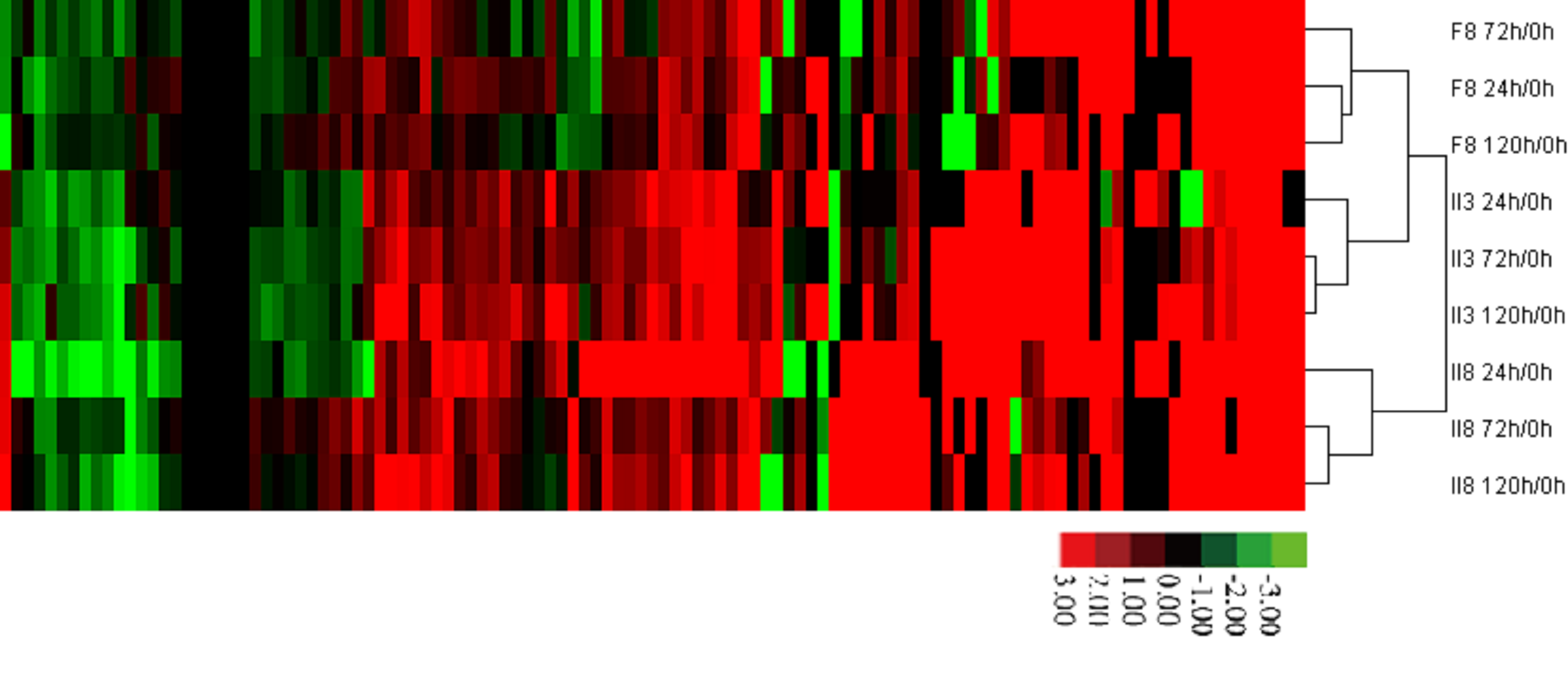

Supplement: Supplemental Information 3 — The color scale indicates of the averages of the normalized values. II8, hybrid rice II YOU 838; F8, paternal Fu Hui 838; maternal II3, II-32A. [file peerj-08-8306-s003.png]
