# Supplementary material for: Transcriptomic and proteomic profiles of II YOU 838 (Oryza sativa) provide insights into heat stress tolerance in hybrid rice
Source: PeerJ. 2020 Feb 21;8:e8306. doi: 10.7717/peerj.8306 (PMC7039125; doi:10.7717/peerj.8306)
Supplement: Supplemental Information 4 [file peerj-08-8306-s004.docx]

**Table S1:**

**Primers used for the quantitative real-time RT-PCR analysis in this study.**

| Gene_ID | RAP_ID | Functional annotation | Forward primer (5’-3’) | Reverse primer (5’-3’) | Amplicon length (bp) |
| --- | --- | --- | --- | --- | --- |
| MH03g0618400 | Os03g0718100 | actin -1 | CAGCCACACTGTCCCCATCTA | AGCAAGGTCGAGACGAAGGA | 67 |
| MH01g0139700 | Os01g0228300 | Mpv17 | GAAGAGGGTGGGCATCACAA | AGACCATCCGCAGCAACTTT | 150 |
| MH02g0177100 | Os02g0267000 | Chloroplast processing peptidase | TTGTCGCCGAGAAGGTTACA | GCAGCACTGGTGGACTTTTG | 81 |
| MH03g0686900 | Os03g0776900 | Mitochondrial import inner membrane translocase subunit TIM14-3 | GGCACTGATCCTTGGCGTAA | GTAATGGCTCCCACCAGCAT | 106 |
| MH05g0501600 | Os05g0519700 | Heat shock transcription factor gene OsHsfA4d | CACGAGCAACTCAGGAAGGT | AGACAGAATGACGTCCAGCG | 114 |
| MH03g0018700 | Os03g0113700 | Heat shock 70 kDa protein | CTCTGGTGGGCTTTCTGAGG | CGATGAGGGCTTTCCGTTCT | 92 |
| MH04g0704000 | Os04g0688100 | Peroxidase 12 | CGAAACCTGGACAAGACGGA | TTCTTCACCAGCACAGGGTC | 128 |
| MH10g0025400 | Os10g0118800 | Probable ascorbate-specific transmembrane electron transporter 1 | TAGCACGCTTCATCTTCCCC | CGAACATGAGGATGGCGAGA | 84 |
| MH03g0762100 | Os03g0854500 | Heat shock transcription factor gene OsHsfA1 | AGGCTGAGACAACAACAGCA | GTTGTTGCCGTTGCTCCATT | 85 |
| MH03g0260900 | Os03g0358000 | HUA2-like protein 2 | TGATAAGTGAGCCAGAGCAGTG | CAGATCCTTTTCCCTGTCGCT | 165 |
| MH07g0536400 | Os07g0677500 | Peroxidase 2 | CGTCTGTTCTGTTGTCGGGA | GCCTCTATCTGCGCCTTGAT | 100 |
