# Supplementary material for: Transcriptomic and proteomic profiles of II YOU 838 (Oryza sativa) provide insights into heat stress tolerance in hybrid rice
Source: PeerJ. 2020 Feb 21;8:e8306. doi: 10.7717/peerj.8306 (PMC7039125; doi:10.7717/peerj.8306)
Supplement: Supplemental Information 6 [file peerj-08-8306-s006.docx]

**Table S3:**

**The markedly differentially expressed over-dominant genes in the flag leaves of II YOU 838 rice after heat treatment.**

| No. | Gene ID | log_2_ FC | | | Functional annotation |
| --- | --- | --- | --- | --- | --- |
|  |  | 24 h/0 h | 72 h/0 h | 120  h/0 h |  |
| 1 | MH01g0081900 | 17.1 | 15.5 | 17.7 | Galactinol--sucrose galactosyltransferase |
| 2 | MH01g0123900 | 18.2 | 13.8 | 16.4 | E3 ubiquitin-protein ligase ATL41 |
| 3 | MH01g0226600 | 17.8 | 0.0 | 12.5 | hypothetical protein |
| 4 | MH01g0323300 | 17.6 | 12.5 | 0.0 | hypothetical protein OsI_02060 |
| 5 | MH01g0328200 | 17.2 | 13.5 | 14.5 | hypothetical protein OsI_02069 |
| 6 | MH01g0365400 | 16.1 | 16.1 | 17.0 | unnamed protein product |
| 7 | MH01g0445400 | 16.5 | 16.6 | 17.8 | hypothetical protein |
| 8 | MH01g0458600 | 17.2 | 0.0 | 0.0 | Vacuolar amino acid transporter 1 |
| 9 | MH01g0488800 | 16.9 | 14.3 | 14.3 | Serine carboxypeptidase-like 50 |
| 10 | MH01g0492100 | -15.1 | 1.3 | 2.0 | Receptor-like protein kinase |
| 11 | MH01g0546600 | 16.9 | 13.1 | 14.1 | MAP3K A |
| 12 | MH01g0581100 | 16.6 | 15.5 | 17.2 | WRKY transcription factor 65 |
| 13 | MH01g0589100 | 17.6 | 12.5 | 15.6 | retrotransposon protein |
| 14 | MH01g0665600 | 3.7 | 2.5 | -13.1 | Pentatricopeptide repeat-containing protein |
| 15 | MH01g0671400 | -15.1 | 1.1 | 2.1 | hypothetical protein OsI_04255 |
| 16 | MH01g0684800 | 16.9 | 15.1 | 16.6 | ZAT8-C2H2 zinc finger protein |
| 17 | MH01g0704500 | 17.6 | 12.5 | 0.0 | hypothetical protein OsI_04531 |
| 18 | MH01g0704800 | 16.7 | 0.0 | 15.8 | MYB family transcription factor |
| 19 | MH01g0791000 | 15.0 | 16.6 | 17.4 | Glutathione S-transferase |
| 20 | MH01g0800100 | 15.6 | 15.8 | 18.5 | Abscisic stress-ripening protein 1 |
| 21 | MH02g0026100 | 15.1 | 14.7 | 17.3 | Serine/threonine-protein kinase HT1 |
| 22 | MH02g0084000 | 16.8 | 13.1 | 12.5 | Uncharacterized protein L728 |
| 23 | MH02g0282700 | 17.6 | 13.5 | 14.8 | Retrovirus-related Pol polyprotein from transposon TNT 1-94 |
| 24 | MH02g0300700 | 17.3 | 14.1 | 13.1 | F-box/kelch-repeat protein |
| 25 | MH02g0392300 | 16.5 | 15.1 | 16.9 | Transcription factor RF2a |
| 26 | MH02g0392700 | 16.9 | 12.5 | 14.6 | Hypothetical protein |
| 27 | MH02g0427800 | 2.6 | 1.6 | -14.5 | Hypothetical protein |
| 28 | MH02g0469800 | 17.2 | 14.8 | 15.4 | Protein phosphatase 2C 16 |
| 29 | MH02g0493300 | 17.8 | 0.0 | 0.0 | Phenylalanine ammonia-lyase |
| 30 | MH02g0493600 | 18.6 | 0.0 | 0.0 | Phenylalanine ammonia-lyase OsPAL4 |
| 31 | MH02g0561400 | 17.2 | 13.1 | 13.1 | Peptide transporter PTR2 |
| 32 | MH02g0595700 | 16.9 | 0.0 | 0.0 | Transcription factor BIM1, bHLH |
| 33 | MH02g0661300 | 17.4 | 0.0 | 0.0 | 18.6 kDa Hsp class III |
| 34 | MH03g0137000 | 17.2 | 13.8 | 14.1 | U-box domain-containing protein 21 E3U-box |
| 35 | MH03g0172200 | -18.0 | 0.2 | 2.4 | Cysteine-rich repeat secretory protein 55 |
| 36 | MH03g0235900 | 4.1 | 0.4 | -13.1 | hypothetical protein OsI_11577 |
| 37 | MH03g0259100 | 4.2 | 1.8 | -12.5 | expressed protein |
| 38 | MH03g0358500 | 18.9 | 0.0 | 14.1 | Zinc finger protein 1 |
| 39 | MH03g0408700 | 16.8 | 0.0 | 13.5 | Transposon Ty3-I Gag-Pol polyprotein |
| 40 | MH03g0687400 | 17.8 | 14.1 | 14.3 | hypothetical protein OsI_13728 |
| 41 | MH03g0745800 | 16.7 | 13.8 | 13.1 | hypothetical protein |
| 42 | MH03g0749700 | 17.0 | 14.1 | 15.2 | Anthocyanidin 5,3-O-glucosyltransferase |
| 43 | MH04g0010600 | 16.9 | 0.0 | 0.0 | protein phosphatase 2C-like |
| 44 | MH04g0555500 | 16.7 | 14.9 | 15.1 | Homeobox-leucine zipper protein HOX22 |
| 45 | MH04g0590800 | 16.8 | 14.3 | 15.6 | Aminocyclopropane-1-carboxylate synthase |
| 46 | MH04g0645800 | 17.6 | 12.5 | 15.3 | hypothetical protein OsJ_16307 |
| 47 | MH05g0063600 | 12.6 | 16.9 | 20.2 | Peroxidase 1 |
| 48 | MH05g0361700 | 17.3 | 12.5 | 13.8 | Pre-mRNA-splicing factor ATP-dependent RNA helicase mog-4 |
| 49 | MH05g0368900 | 16.7 | 14.3 | 14.8 | Kinesin-4 |
| 50 | MH05g0399700 | 19.8 | 13.5 | 0.0 | Phenylalanine ammonia-lyase |
| 51 | MH05g0427800 | 16.9 | 13.5 | 14.3 | MYB family transcription factor 3R-1 |
| 52 | MH05g0463700 | 14.1 | 13.5 | 16.6 | OSJNBa0038P21.8 |
| 53 | MH05g0465000 | 17.2 | 12.5 | 14.3 | unnamed protein product |
| 54 | MH06g0146900 | 17.0 | 12.5 | 12.5 | 26.2 kDa Hsp |
| 55 | MH06g0492400 | 16.8 | 13.5 | 13.8 | Tetrahydrocannabinolic acid synthase |
| 56 | MH06g0583000 | -13.5 | 2.1 | 3.7 | unknown protein |
| 57 | MH06g0689400 | 18.1 | 13.5 | 15.2 | Activator of 90 kDa heat shock protein ATPase homolog 2 |
| 58 | MH07g0015600 | 4.3 | 0.5 | -12.5 | Dirigent protein 1 |
| 59 | MH07g0428300 | 5.6 | 1.5 | -12.5 | Alternative NAD(P)H dehydrogenase 1 |
| 60 | MH07g0434600 | 16.8 | 13.1 | 13.5 | L-type lectin-domain containing receptor kinase IX.1 |
| 61 | MH07g0501100 | 3.8 | -13.1 | -0.6 | CBL-interacting protein kinase 21 |
| 62 | MH08g0051700 | 17.3 | 0.0 | 12.5 | Phospholipase A1-Ibeta2 |
| 63 | MH08g0529300 | 16.9 | 15.2 | 15.6 | Transcription initiation factor IIB 1 |
| 64 | MH09g0309700 | 17.2 | 14.7 | 15.2 | MYB family transcription factor |
| 65 | MH09g0352600 | 6.2 | -12.5 | 2.6 | GAMETE EXPRESSED 1 |
| 66 | MH09g0354400 | 18.2 | 16.0 | 18.0 | plant viral-response family protein-like |
| 67 | MH10g0061500 | 17.6 | 0.0 | 13.5 | retrotransposon protein |
| 68 | MH10g0188800 | 17.6 | 0.0 | 13.8 | hypothetical protein |
| 69 | MH10g0331700 | 15.3 | 16.1 | 18.6 | Beta-amylase 1 |
| 70 | MH10g0392800 | 17.1 | 14.1 | 16.4 | Glutathione S-transferase GSTU6 |
| 71 | MH11g0017000 | 2.8 | -13.8 | -0.4 | PRA1 family protein B5 |
| 72 | MH12g0067800 | 16.7 | 16.0 | 13.5 | Hypothetical protein |
| 73 | MH12g0101700 | 0.0 | 14.9 | 19.4 | Carboxyvinyl-carboxyphosphonate phosphorylmutase |
