# Supplementary material for: Transcriptomic and proteomic profiles of II YOU 838 (Oryza sativa) provide insights into heat stress tolerance in hybrid rice
Source: PeerJ. 2020 Feb 21;8:e8306. doi: 10.7717/peerj.8306 (PMC7039125; doi:10.7717/peerj.8306)
Supplement: Supplemental Information 9 [file peerj-08-8306-s009.docx]

**Table S6:**

**Transcription factor genes that were highly differentially expressed in hybrid rice II YOU 838 flag leaves** **under long-term heat stress.**

| No. | Gene ID | log2 FC | | | Transcription factors family | Functional annotation |
| --- | --- | --- | --- | --- | --- | --- |
|  |  | 24 h/0 h | 72 h/0 h | 120h/0 h |  |  |
| 1 | MH09g0359900 | 4.4 | -10.5 | 0 | Alfin-like | PHD finger protein MALE STERILITY 1 |
| 2 | MH01g0056500 | 2.7 | 2.6 | 2.4 | AP2-EREBP | AP2/ERF and B3 domain-containing protein |
| 3 | MH04g0622600 | 4.2 | 1 | 3.6 | AP2-EREBP | AP2/ERF, OsAP2-39 |
| 4 | MH10g0429500 | 5 | 1.2 | 1.7 | AP2-EREBP | Ethylene-responsive transcription factor 5 |
| 5 | MH01g0017100 | -3.8 | 0.8 | -0.4 | bHLH | bHLH36 |
| 6 | MH01g0017400 | -2 | 0.2 | -0.2 | bHLH | bHLH8 |
| 7 | MH03g0648500 | 3.1 | 0.4 | 0.4 | bHLH | bHLH92 |
| 8 | MH03g0690900 | -0.4 | -2.5 | -2.1 | bHLH | bHLH |
| 9 | MH08g0482900 | 3.1 | 2.1 | 1.8 | bHLH | Transcription factor BIM2 |
| 10 | MH02g0034500 | -3.1 | 0.4 | 0.4 | bZIP | Transcription factor HBP-1a |
| 11 | MH06g0591100 | 2.6 | 0.5 | 1.9 | bZIP | bZIP transcription factor 60 |
| 12 | MH07g0509000 | 3.1 | 1.4 | 2.4 | bZIP | OsbZIP60 |
| 13 | MH12g0424100 | -0.4 | -2 | -3.1 | bZIP | Light-inducible protein CPRF2 |
| 14 | MH02g0592600 | -3.8 | 0.1 | -0.1 | C2C2-CO-like | Zinc finger protein CONSTANS-LIKE 9 Days to heading |
| 15 | MH08g0182400 | 4.1 | 0.1 | 2.6 | C2C2-CO-like | Zinc finger protein CONSTANS-LIKE 3 |
| 16 | MH03g0074500 | -0.9 | -2.6 | -3 | C2C2-Dof | Cyclic dof factor 2 Dof Transcription Factor |
| 17 | MH02g0689800 | 3.2 | 0.2 | 2 | C2C2-GATA | GATA transcription factor 11 |
| 18 | MH06g0708300 | -3.1 | -1 | -1.6 | C2C2-YABBY | DNA-binding protein MNB1B High-mobility group box HMGB1 |
| 19 | MH03g0733200 | 6.7 | 2.6 | 4.5 | C2H2 | Zinc finger protein ZAT12 |
| 20 | MH08g0249500 | 3.3 | -0.9 | 1.8 | C2H2 | Zinc finger protein 1 |
| 21 | MH01g0680900 | 2.8 | 1.1 | 1.3 | C3H | Zinc finger CCCH domain-containing protein 11 |
| 22 | MH09g0403500 | 2.1 | 0.9 | 0.9 | C3H | Zinc finger CCCH domain-containing protein 11 |
| 23 | MH06g0213800 | 2.7 | 1.5 | 1.7 | CPP | Histone-lysine N-methyltransferase EZ1 ESC-like gene |
| 24 | MH06g0554300 | 1.2 | 1.5 | 3.3 | FAR1 | Protein FAR1-RELATED SEQUENCE 5 |
| 25 | MH09g0412200 | 2.1 | 0.7 | 0.9 | FHA | Fork head transcription factor 1 |
| 26 | MH11g0036100 | -3.6 | -0.6 | -2 | FHA | Zeaxanthin epoxidase |
| 27 | MH12g0038500 | -4 | -1.2 | -2 | FHA | Zeaxanthin epoxidase |
| 28 | MH05g0501600 | 3.4 | 0.5 | 1.6 | HSF | Heat stress transcription factor A-4d |
| 29 | MH07g0091300 | 4.1 | 2.4 | 1.9 | HSF | Heat stress transcription factor A-2b |
| 30 | MH09g0368500 | 2.5 | 1.9 | 3.1 | HSF | Heat stress transcription factor B-1 |
| 31 | MH07g0460500 | 2 | 1.2 | 2.6 | LOB | LOB domain-containing protein 37 |
| 32 | MH12g0013900 | 14.5 | 15.7 | 16.8 | LOB | LOB domain-containing protein 12 |
| 33 | MH02g0600000 | -2.7 | -2 | -1.5 | MADS | MADS-box transcription factor 57 |
| 34 | MH04g0379200 | 2.1 | 0.6 | -0.8 | MADS | MADS-box transcription factor 18 |
| 35 | MH07g0474300 | -2.1 | -1.4 | -1.2 | MADS | MADS-box transcription factor 18 |
| 36 | MH02g0242700 | 0.7 | 2.3 | 1.7 | MYB | GARP-type Transcriptional Repressor 1 NIGT1 |
| 37 | MH02g0674900 | 2.4 | 0.6 | -14.2 | MYB | Glutathione S-transferase T3 |
| 38 | MH02g0359900 | -0.7 | 1.4 | 2.9 | MYB-related | 11-beta-hydroxysteroid dehydrogenase 1B |
| 39 | MH07g0545300 | 16.7 | 13.6 | 15.1 | MYB-related | Myb-related protein 305 |
| 40 | MH08g0066700 | -3.1 | -2.7 | -2.8 | MYB-related | Protein LHY |
| 41 | MH08g0419700 | 2.9 | 2.8 | 3.7 | MYB-related | Protein ODORANT1 |
| 42 | MH09g0309700 | 15.9 | 13.1 | 14.3 | MYB-related | Protein ODORANT1 |
| 43 | MH01g0788700 | 1.5 | 2.2 | 2.1 | NAC | Protein SOMBRERO |
| 44 | MH02g0443600 | -1.4 | 0.4 | 2.5 | NAC | NAC domain-containing protein 100 |
| 45 | MH03g0687300 | 2.3 | 1.2 | 0.8 | NAC | Transcription factor JUNGBRUNNEN 1 |
| 46 | MH03g0728200 | 4 | 1.8 | 2.7 | NAC | NAC domain-containing protein 67 |
| 47 | MH07g0041200 | 15.5 | 14.1 | 13.7 | NAC | Transcription factor JUNGBRUNNEN 1 |
| 48 | MH07g0137200 | 4 | 2 | 2.3 | NAC | NAC domain-containing protein 67 |
| 49 | MH07g0540800 | -1.8 | 0.7 | 3.2 | NAC | NAC transcription factor 29 |
| 50 | MH11g0035600 | 0 | 1.6 | 4.7 | NAC | NAC domain-containing protein 77 |
| 51 | MH11g0035900 | 0 | 0 | 15.7 | NAC | NAC domain-containing protein 77 |
| 52 | MH12g0038200 | 4.6 | 3.7 | 6.2 | NAC | NAC transcription factor ONAC010 |
| 53 | MH12g0038300 | 1.5 | 1.5 | 5.2 | NAC | NAC domain-containing protein 77 |
| 54 | MH12g0309900 | 2.5 | 0.9 | 1.7 | NAC | NAC transcription factor 29 |
| 55 | MH06g0056600 | -2.7 | 0.3 | -0.5 | PBF-2-like | Single-stranded DNA-binding protein WHY1 |
| 56 | MH09g0457700 | -2.1 | -0.1 | -1.7 | RWP-RK | Protein NLP1 |
| 57 | MH02g0045300 | -2.8 | -1.7 | -2.4 | SBP | Squamosa promoter-binding-like protein 3 |
| 58 | MH04g0495100 | 4.2 | 1.9 | 4.8 | TAZ | BTB/POZ and TAZ domain-containing protein 1 |
| 59 | MH12g0436400 | -2.6 | -0.3 | -0.8 | TCP | Transcription factor PCF8 |
| 60 | MH05g0034700 | 5.9 | 3.8 | 5.7 | Trihelix | Trihelix transcription factor GT-3b |
| 61 | MH05g0528700 | 2.5 | 0.9 | 1.2 | TUB | Tubby-like F-box protein 10 |
| 62 | MH01g0445100 | 3 | 1.5 | 0.4 | WRKY | WRKY transcription factor 50 |
| 63 | MH05g0306800 | 2.7 | 0.9 | 1.8 | WRKY | WRKY transcription factor 33 |
| 64 | MH01g0668900 | 4.3 | 2.6 | 1.9 | WRKY | WRKY transcription factor 46 |
| 65 | MH01g0673800 | 4 | 2.2 | 3.6 | WRKY | WRKY transcription factor 33 |
| 66 | MH02g0085100 | 4 | 0.9 | 2.7 | WRKY | WRKY transcription factor 40 |
| 67 | MH03g0212600 | 1.8 | 1.3 | 2.2 | WRKY | WRKY transcription factor 46 |
| 68 | MH03g0223300 | 1.3 | 2.2 | 3 | WRKY | WRKY transcription factor 70 |
| 69 | MH04g0232500 | 2.4 | 1.5 | 1.9 | WRKY | WRKY transcription factor 11 |
| 70 | MH05g0036100 | -4.1 | -3.3 | -2.2 | WRKY | WRKY transcription factor 65 |
| 71 | MH05g0287900 | 15.5 | 0 | 12.9 | WRKY | WRKY transcription factor 55 |
| 72 | MH05g0538600 | 1.7 | 2.2 | 2.8 | WRKY | WRKY transcription factor 41 |
| 73 | MH05g0550100 | 0 | 1.1 | 4.1 | WRKY | WRKY transcription factor 22 |
| 74 | MH06g0077400 | 2.2 | 0.5 | 2.7 | WRKY | WRKY transcription factor 53 |
| 75 | MH08g0380600 | 3.3 | 1.9 | 3 | WRKY | WRKY transcription factor 53 |
| 76 | MH09g0241500 | 2.2 | 1.6 | 2.6 | WRKY | WRKY transcription factor 46 |
| 77 | MH12g0033600 | 0.9 | 2.9 | 3.9 | WRKY | WRKY transcription factor 70 |
